# Removal of chemical and microbial water pollutants by cold plasma combined with Ag/TiO_2_-rGO nanoparticles

**DOI:** 10.1038/s41598-022-13444-2

**Published:** 2022-06-14

**Authors:** Mahmoud S. Abdel-Wahed, Mohamed Mokhtar Hefny, Sherif Abd-Elmaksoud, Mohamed A. El-Liethy, Marwa A. Kamel, Amer S. El-Kalliny, Ibrahim Ahmed Hamza

**Affiliations:** 1 Water Pollution Research Department, National Research Centre, 33 El Buhouth St., Dokki, Giza, 12622 Egypt; 2 Engineering Mathematics and Physics Department, Faculty of Engineering and Technology, Future University in Egypt, Cairo, Egypt

**Keywords:** Biophysics, Environmental sciences, Chemistry

## Abstract

This study aimed to investigate the synergistic effect of the cold atmospheric plasma (CAP) and heterogeneous photocatalytic processes in an aqueous solution to enhance water purification efficacy and reduce the energy cost required by CAP. 0.1% Ag/TiO_2_-reduced graphene oxide (rGO) nanoparticles (NPs) photo-composite were prepared and fully characterized. Data showed that Ag nanoparticles and the rGO play an important role in increasing the efficiency of the whole treatment process and the photo-composite **(**0.1% Ag/TiO_2_-1% rGO at 400 °C) revealed the highest phenol removal rate with excellent reusability. Also, complete inactivation (~ 5log_10_ reduction) of both *E. coli* and *S. aureus* by NPs was observed without CAP exposure, whereas a minimal effect (0.1–0.5 log_10_) on viruses (Adenovirus (AdV), rotavirus, and ɸX174) was observed after 10 min incubation. Interestingly, the photocatalytic virus inactivation test was promising, as it resulted in > 4.7log_10_ reduction of AdV at 2 min treatment, whereas < 1log_10_ could be reduced using only CAP at the same treatment time. Accordingly, we believe that this work could provide new insights into how the synergy between CAP and 0.1% Ag/TiO_2_-1% rGO photo-composite in aqueous media imposes a great potential for environmental applications, such as water purification and microbial inactivation.

## Introduction

There is a public health threat posed by water pollution due to chemical and pathogenic microorganisms in water. The cold (non-thermal) atmospheric plasma (CAP) is capable of producing high densities of reactive oxygen species (ROS) and reactive nitrogen species (RNS)^1^. These reactive oxygen and nitrogen species are generated because of the very energetic electrons in CAP, where electrons generate high densities of internally excited species (metastables) or radicals in inelastic electron-impact excitation and dissociation collisions, respectively. We have recently evaluated the efficacy of CAP in the removal of both chemical and microbial pollutants in water^2^. However, further enhancement of the water purification efficacy of CAP can be achieved by the synergistic action of plasma and photocatalytic processes which can also reduce the energy cost^3–5^.

A heterogeneous photo-oxidation process effectively using TiO_2_ for removal of organic water pollutants^3,4,6,7^ and disinfection^5,7^ was reported. Mainly, TiO_2_ is inexpensive and has been combined with plasma because it has a strong oxidation power, a reasonable bandgap, nontoxicity, and stability against photo- and chemical-corrosions^8^. However, TiO_2_ can be activated only in the UV part of the plasma spectrum. For instance, in the case of anatase TiO_2_, the bandgap is 3.2 eV, therefore UV light (λ ≤ 385 nm) is required for the activation process^5^. Therefore, improving TiO_2_ photocatalyst is necessary to invest in the visible region of the plasma spectrum and to increase the efficiency of the process as a whole (the optical emission spectrum of CAP is usually presented from approximately 300 nm to 800 nm with the major reactive species^9^. The enhancement of the photocatalytic activity of TiO_2_ photocatalyst is not only due to the modification of band energy but also due to the prolonged lifetime of free charge carriers (i.e., charge separation). Doping with noble metal nanoparticles (NPs) with surface plasmon resonance (SPR) such as Ag promotes the generation of electron–hole (e^−^/h^+^) pairs, where the collective oscillation of free conduction electrons on the surface of Ag metal can facilitate the separation of electrons and holes generated on the surface of the TiO_2_ semiconductor^10^. In addition, it increases light scattering to capture a greater portion of light for coupled TiO_2_ semiconductors^11^. Recently, Ag/TiO_2_ composite was reported to have a high separation rate of electron and hole, which enhanced the degradation of phenol when combined with CAP^12^.

On the other hand, Ag NPs have antimicrobial activity and biocompatibility nanomaterials and have been used for water disinfection^13^. The physically perturbing action of Ag NPs induces oxidative stress, disrupting a microbial process via the oxidization of one of the components of the cell membrane, or a disruption of a process that takes place inside the cell^14^. TiO_2_ has caught the attention of many researchers due to its ability to inactivate a wide variety of viruses^15,16^. However, doping of TiO_2_ with Ag NPs was used to enhance the antimicrobial activity due to their synergistic effect^17^.

In addition, the role of reduced graphene oxide (rGO) in the enhancement of TiO_2_ photocatalytic activity was studied before^8,11,18^. The improvement of the photocatalytic activity is mainly due to decreasing the recombination of e^−^/h^+^ pairs of TiO_2_ via withdrawing the excited electrons by the π-π bond in rGO^19,20^. Moreover, rGO shifts the TiO_2_ excitation range into the visible region^8,19,20^.

While the photocatalytic activity of Ag/TiO_2_-rGO photo-composite in the presence of light was covered elsewhere^11,13,18,21^, to the best of our knowledge the combination and activation of this composite with CAP source and its antimicrobial activity were not investigated to date. Thus, this study addresses this scientific research gap. In this work, the synergism between the CAP and Ag/TiO_2_-rGO photo-composite for chemical and microbial removal from aqueous media was investigated. Phenol was used as a model of water organic micropollutant compound. In order to study the photocatalytic microbial inactivation capabilities of 0.1% Ag/TiO_2_-1% rGO photo-composite, *E. coli* and *S. aureus* were used as a model of Gram-negative and Gram-positive bacteria; respectively. Since enteric viruses are relatively more resistant to water disinfection compared to bacteria, the effect of CAP and 0.1% Ag/TiO_2_-1% rGO on adenovirus (AdV), rotavirus (RoV), and ɸX174 was also explored. Besides, the mechanism of the main formed reactive species through this process with their contribution to the chemical and microbial removal was suggested using radical scavengers.

## Results and discussion

### Characteristics of the prepared photocatalysts

**Figure 1:**
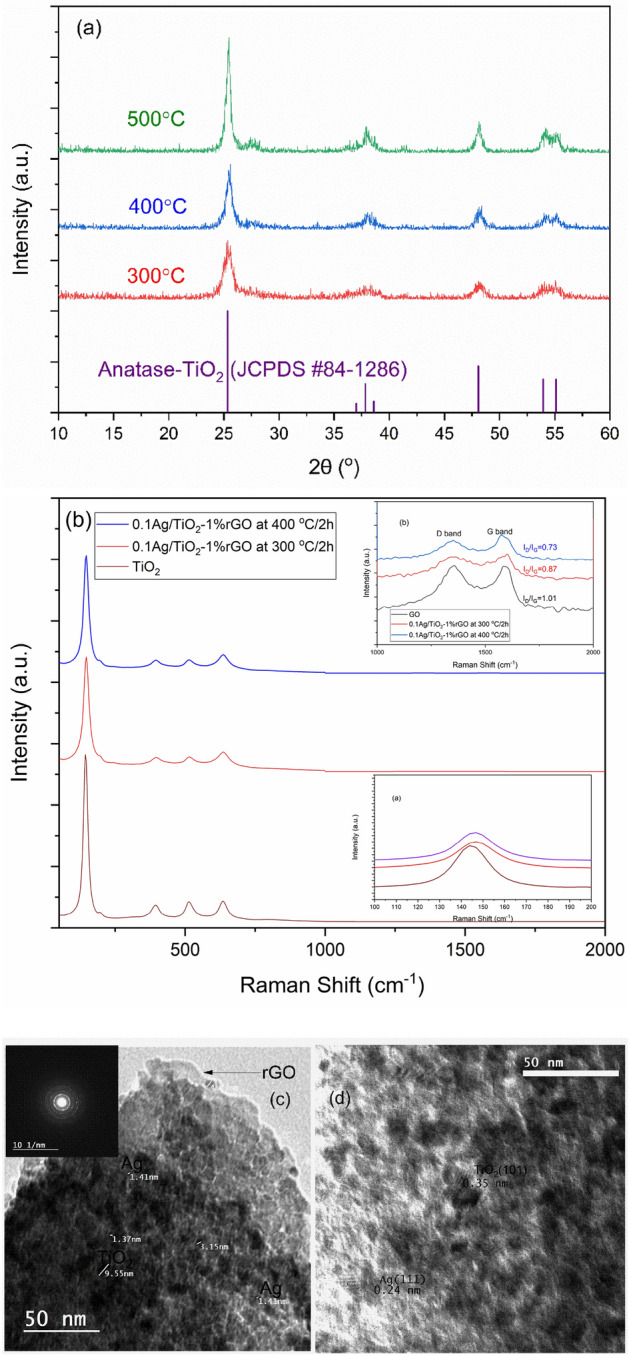
(**a**) XRD pattern, (**b**) Raman spectra of 0.1%Ag/TiO_2_-1%rGO at different calcination temperatures. (**c, d**) HR-TEM images of 0.1%Ag/TiO_2_-1%rGO at 400 °C for 2 h.

The phase composition of prepared nano-materials was identified by XRD. Figure 1a demonstrated the XRD diffraction pattern for 0.1%Ag/TiO_2_-1%rGO at different calcination temperatures (300 °C, 400 °C and, 500 °C for 2 h). All samples at different calcination temperatures have diffraction patterns at 2 θ of 25.325°, 37.841°, 48.074°, 53.952°, and 55.106° equivalent well with JCPDS card No. 84–1286. It should be noted that all samples showed only the distinctive diffraction patterns of TiO_2_ due to the low content of Ag NPs and rGO sheets according to the amount of TiO_2_. Also, an insufficient number of planes for the production of clear XRD patterns, or the low intensity of single rGO or Ag are in agreement with^8,22^. Therefore, Raman spectroscopy was used to prove the presence of rGO in the prepared photo-composite. On the other hand, the average grain crystal size was calculated according to Scherrer Equation^23^. The average grain crystal size was 6.7, 10.5, and 12.5 nm for samples that were calcinated at 300 °C, 400 °C, and 500 °C, respectively.

In Fig. 1b, the Raman spectra of GO and 0.1%Ag/TiO_2_-1%rGO at calcination temperatures of 300 °C and 400 °C are shown. For the photo-composite, distinct bands of anatase TiO_2_ appeared at 148 cm^−1^, 397 cm^−1^, 514 cm^−1^, and 638 cm^−1^. These bands are associated with the E_g_ optical Raman mode, B1_g_, A1_g_, and E_g_ Raman modes of anatase TiO_2_, respectively. In addition, in Fig. 1b (inset (a)), the TiO_2_ anatase E_g_ mode was also observed to shift from 144 cm^−1^ (pure TiO_2_) to 148 cm^−1^ (0.1%Ag/TiO_2_-1%rGO) owing to the interaction of the metal atoms in TiO_2_ with the rGO sheets. This behavior was observed also by^24^.

Also, D and G bands that are characteristic of GO and rGO are presented with samples of pure GO and 0.1%Ag/TiO_2_-1%rGO calcinated at 300 °C and 400 °C (see Fig. 1b inset (b)). These bands are assigned to disorder carbon (D band) and sp^2^ hybridized carbon (G band). D-bands appeared at 1351 cm^−1^, 1363 cm^−1^, and 1351 cm^−1^, while G bands at 1584 cm^−1^, 1584 cm^−1^, and 1577 cm^−1^ for pure GO, 0.1%Ag/TiO_2_-1%rGO calcinated at 300 °C and 0.1%Ag/TiO_2_-1%rGO calcinated at 400 °C, respectively. There is a marginal shift ∼7 cm^−1^ to a lower frequency at 1577 cm^−1^ (0.1%Ag/TiO_2_-1%rGO calcinated at 400 °C) from 1584 cm^−1^ (pure GO), while there is no shift in the case of the photo-composite calcinated at 300 °C. This confirms the charge transfer between TiO_2_ and rGO when the calcination temperature increases to 400 °C^18^. Therefore, it is expected to find a better photocatalytic performance of the photo-composite that was calcinated at 400 °C compared to that calcinated at 300 °C. This phenomenon will be clarified later in the photocatalytic performance section. The intensity ratio I_D_/I_G_ is 1.01, 0.87, and 0.73 for pure GO, 0.1%Ag/TiO_2_-1%rGO calcinated at 300 °C, and 400 °C, respectively. This is due to deoxygenated of the GO group at 300 °C and 400 °C and reconstructed π–π conjugated structure in rGO. So that, the decrease in the intensity ratio of I_D_/I_G_ is due to the reduction of GO to rGO^8,25^. Also, no Raman peaks for Ag were showed due to the crystal symmetry of Ag.

The morphology of the photo-composite (0.1%Ag/TiO_2_-1%rGO at 400 °C for 2h) was demonstrated by HR-TEM (Fig. 1c, d). There is a good distribution of Ag/TiO_2_ particles over the rGO sheets (Fig. 1c). The selected area electron diffraction (SAED) in the inset of Fig. 1c shows clear circle dots, which indicates that the photo-composite (0.1%Ag/TiO_2_-1%rGO at 400 °C for 2h) is polycrystalline in nature^24^. The SAED ring dot is corresponding to (1 0 1), (0 0 4), and (2 2 0) planes of TiO_2_ anatase, which is in agreement with the XRD pattern (see Fig. 1a). The d-spacing of Ag and TiO_2_ lattice crystal are demonstrated in Fig. 1d.

**Figure 2:**
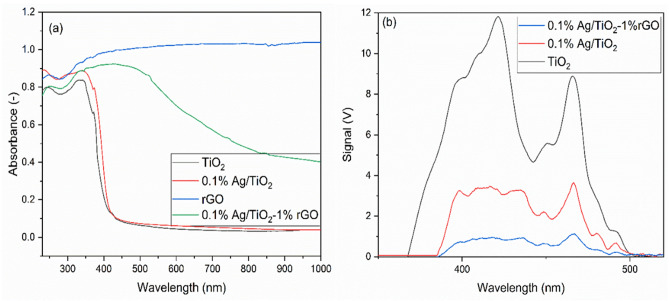
(**a**) UV–Vis absorption spectra and (**b**) PL spectra of the prepared materials (calcinated at 400 °C for 2h) .

The optical properties of the prepared photo-composites were identified by UV–Vis DRS. The UV–Vis absorption spectra of TiO_2_, rGO, 0.1% Ag/TiO_2_, and 0.1% Ag/TiO_2_-1% rGO are presented in Fig. 2a. As appeared in Fig. 2a, the absorption of TiO_2_ is below 400 nm wavelength. There is a little increase in the absorption at  wavelengths longer than 400 nm by adding Ag particles to the TiO_2_. This is due to the effect of incorporation of metallic Ag nanoparticles with TiO_2_, which gives a strong localized surface plasmon resonance (LSPR)^26^. This phenomenon will enhance the photocatalytic performance of TiO_2_ photocatalyst, by decreasing the recombination rate of photo-excited e^−^/h^+^ pairs. On the other hand, the pure rGO has the highest absorption within the spectrum region. Therefore, the 0.1% Ag/TiO_2_-1% rGO photo-composite exhibits light absorption not only in the UV range but also in the visible light range and the change in light absorption properties is mainly due to the presence of 1% rGO. Hence, the photo-composite can absorb all light spectrum introduced by CAP, which increases its reactivity and hence its efficiency for water treatment together with CAP.

Figure 2b shows the PL spectra of TiO_2_, 0.1% Ag/TiO_2_, and 0.1% Ag/TiO_2_-1%rGO. The pure TiO_2_ has the highest broadband emission intensity from 350–500 nm. This is due to the high e^−^/h^+^ pairs recombination rate. This broadband emission intensity was decreased in the case of 0.1% Ag/TiO_2_ as the metallic Ag NPs incorporate in TiO_2_ decreased the e^−^/h^+^ pairs recombination rate. The rGO in the photo-composite (0.1% Ag/TiO_2_-1%rGO) decreases the broadband emission intensity because it can consume the excited electrons by its π-π bonds. This leads to the highest photocatalytic activity as the rate of e^−^/h^+^ pairs recombination is the lowest. This is supporting to the high photocatalytic activity of the prepared photo-composites in the photocatalytic performance section.

### Characteristics of CAP

**Figure 3:**
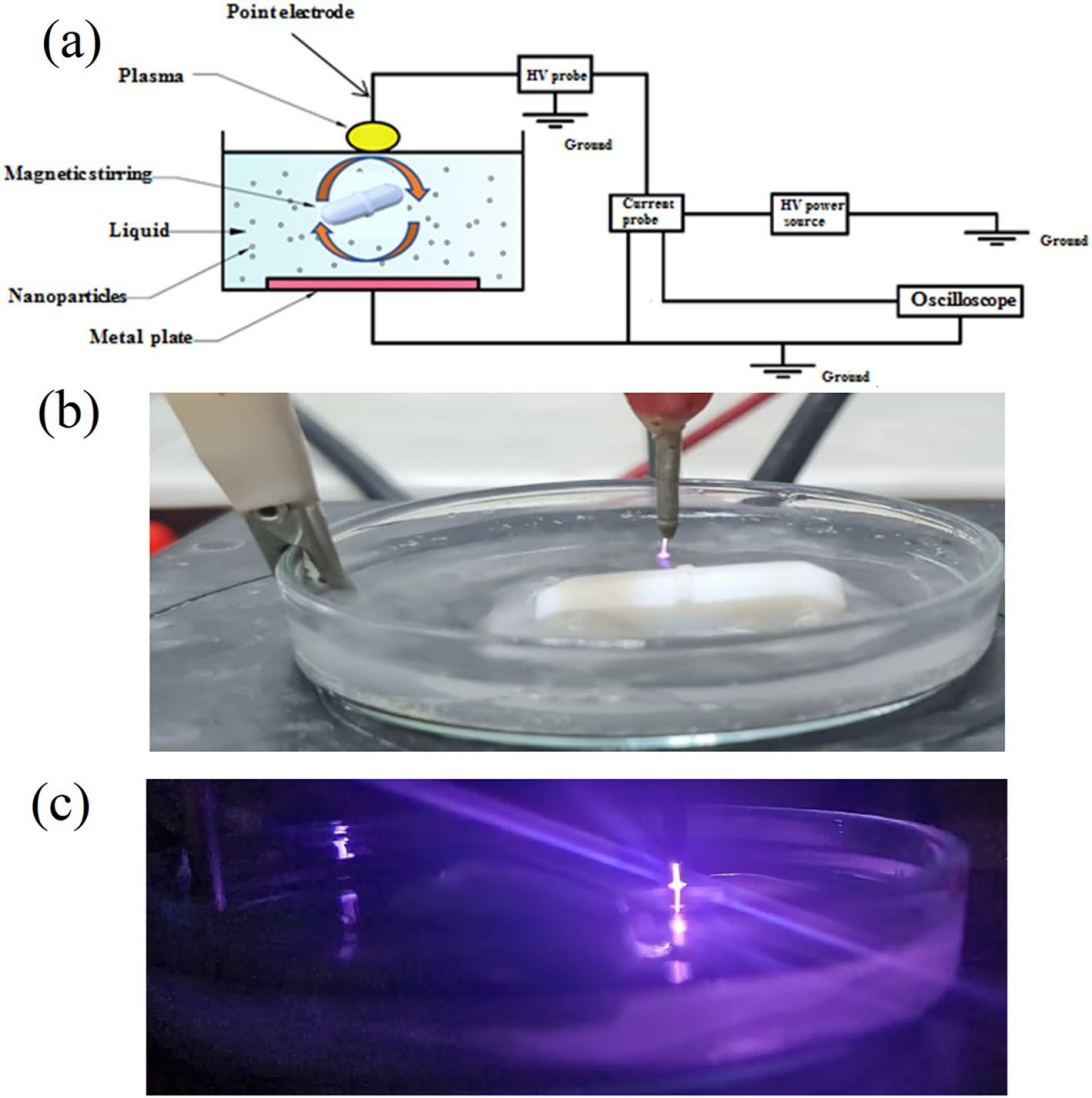
(**a**) Scheme of the plasma reactor used for water treatment. (**b**) A photograph of CAP source (point electrode) during water treatment. (**c**) A photograph of the electrical discharge plasma in dark.

The plasma reactor, as shown in Fig. 3a, consists of an AC high voltage power supply with input voltage ranging from 0 to 18 V and output voltage up to 10 kV, where its positive terminal was connected to the point electrode (above the water surface), while its negative terminal was connected to the metal electrode (platinum) immersed in the treated liquid. All the treatments were performed at optimum operating conditions, which were reached in previous work, at plasma voltage 3 kV peak-to-peak and plasma current 1550 μA (approximately), gap distance of 1 mm, and water thickness of 3.6 mm (see Fig. 3b, c), more details of the system can be found at^2^.

**Figure 4:**
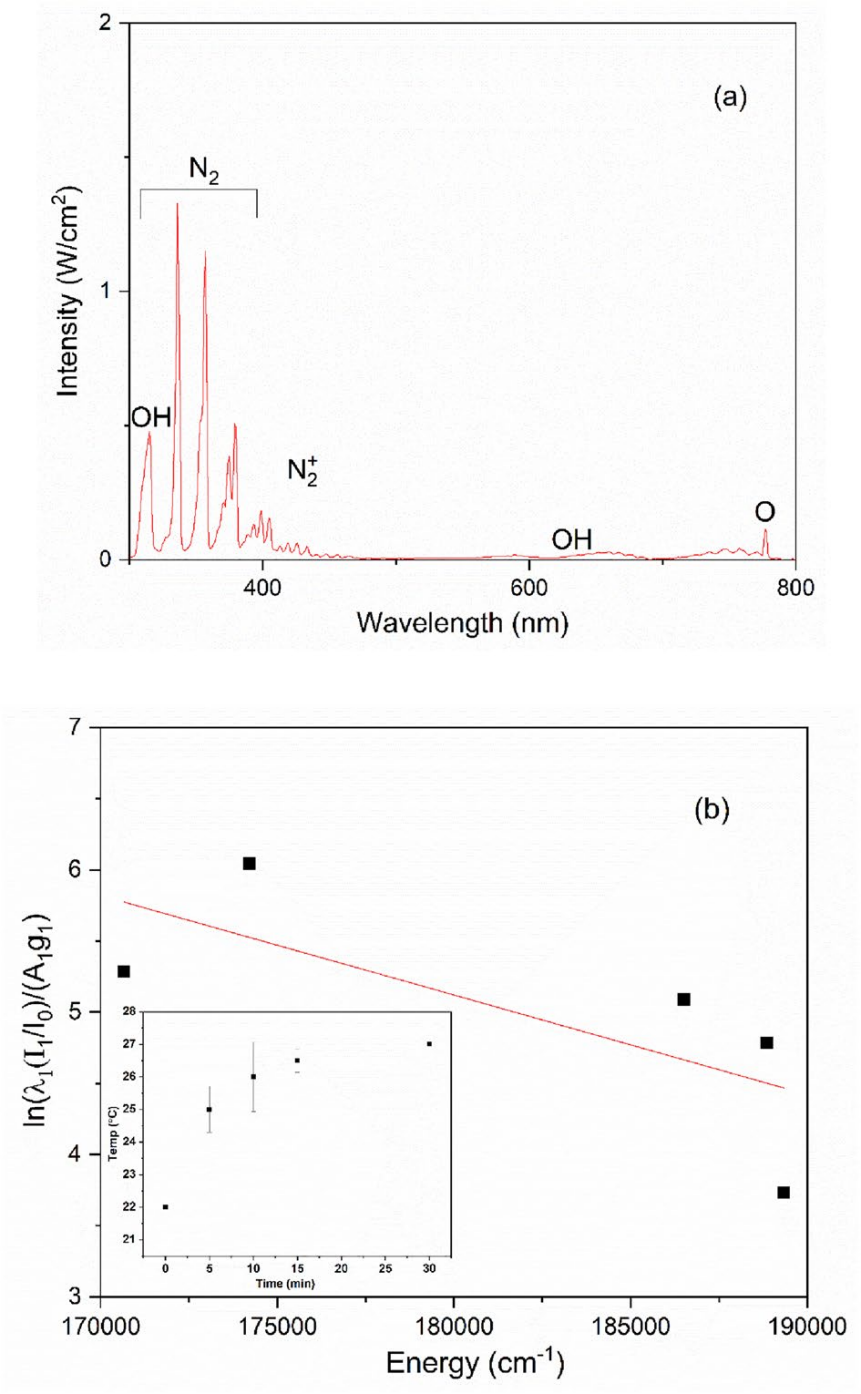
(**a**) Optical emission spectrum of CAP above phenol during the treatment of phenol. (**b**) Boltzmann plot used for estimating electron temperature of the plasma and inset shows the temperature of solution.

The optical emission spectrum (OES) of CAP above phenol was measured during the treatment (see Fig. 4a). Most of the observed lines are in the near-UV region as a result of molecular nitrogen bands (N_2_), nitrogen molecular ions (N_2_^+^), and OH excited species emissions, where N_2_ can be observed from 306 to 380 nm, N_2_^+^ can also be seen from 391 to 470 nm, and OH band between 306 and 317 nm was observed. Moreover, O atoms can be noted at 377 nm^27–30^. From the plasma OES, it was found that RNS can be formed in the gas phase and would be introduced to the plasma-activated medium. Therefore, the formed nitrates at 15 min in the CAP-0.1% Ag/TiO_2_-1%rGO system were 88.7 mg/L and the nitrites were below the detection limit (< 0.001 mg/L) due to the fast conversion to nitrates within this reaction period^31^.

The Boltzmann plot method is used to estimate the plasma electron temperature of our source through the emission spectra of N_2_^+^. The Boltzmann plot utilizes several emission lines to get a good estimation of electron temperature. Therefore, the plasma electron temperature can be measured from the following equation:

1 $$\log \left( { \frac{{(I_{nm} \left( 2 \right)/I_{nm} \left( 1 \right))/\lambda_{nm} }}{{g_{m} \left( 2 \right)A_{nm} \left( 2 \right)}} } \right) = \frac{ - 0.625}{T}E\left( 2 \right) + C,$$

where $I_{nm} \left( 1 \right)$ is the emission intensity of the first line (which is selected as a base), $I_{nm} \left( 2 \right)$ is the emission intensity of the emitted upper level, $g_{m} \left( 2 \right)$ is the statistical weight of the upper level of the transition, $A_{nm} \left( 2 \right)$ is the atomic transition probability, $\lambda_{nm}$ is the wavelength of the emitted upper level (nm), $E\left( 2 \right)$ is the excitation energy of the emitted upper level (cm^-1^), T is the electron temperature (°K), and C is a constant^32–35^. Using this method, five spectral lines of N II were chosen from the spectral lines to determine the electron temperature (see Table S1 in the supporting information (SI)). Figure 4b shows the Boltzmann plot of the above equation with E on the horizontal axis and $\ln \left( { \frac{{(I_{1} /I_{0} )/\lambda_{nm} }}{{A_{1} g_{1} }} } \right)$ on the vertical axis. The electron temperature was calculated from the slope of the fitted line and it was about 6191 (°K), this temperature (measured at plasma voltage 3 kV peak-to-peak) is in the normal range of electron temperature of CAP^32,36,37^. Although the electrons are very energetic as we can see, the heavy particles would be at the room temperature and this is one of the most important advantages of CAP, which make it also suitable for many biomedical application^31^. The temperature of the treated solution was also measured during the whole treatment time with minimal change (≈5 °C) as shown in inset of Fig. 4b.

### Photocatalytic/CAP performance and phenol degradation

**Figure 5:**
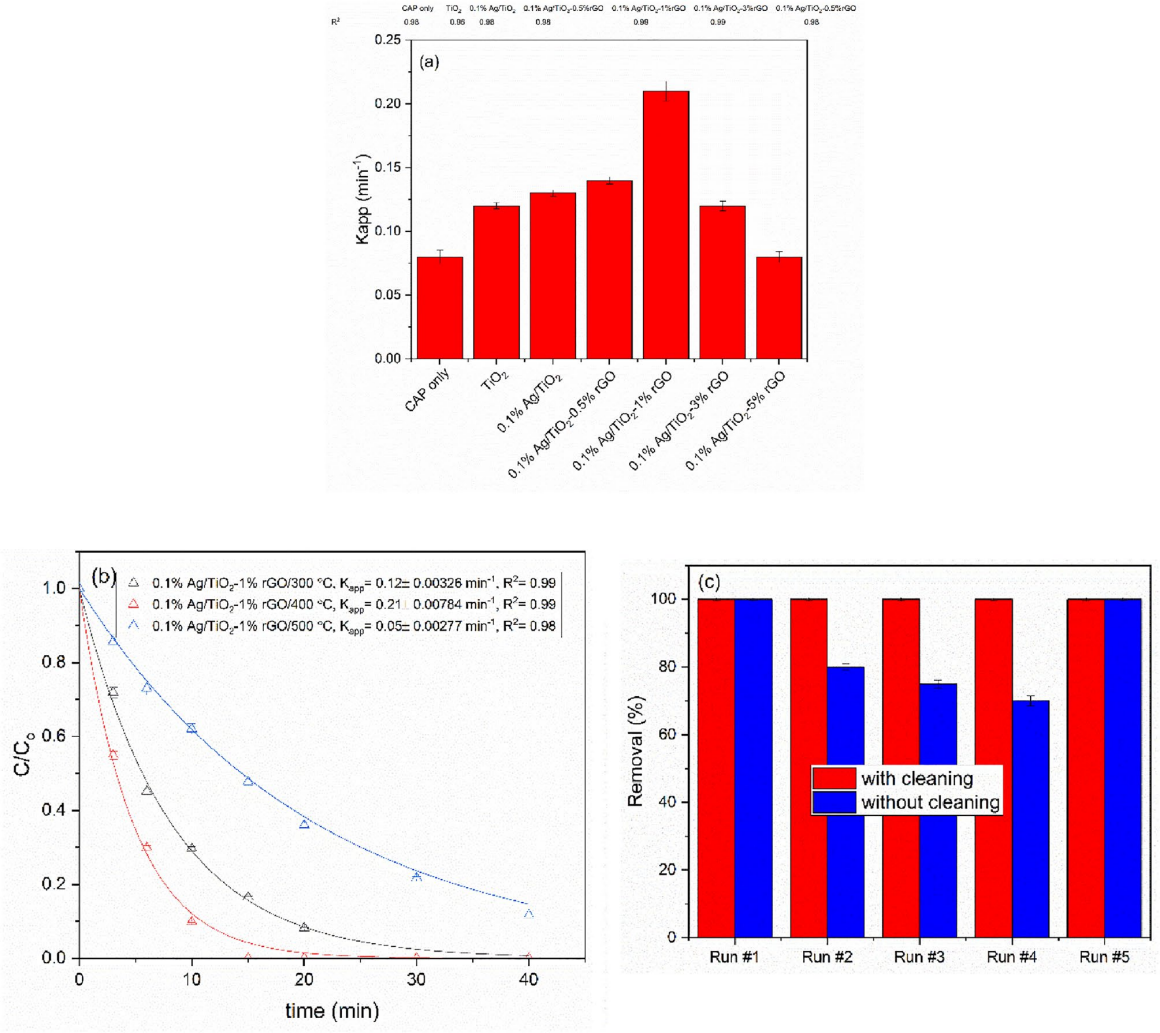
(**a**) The apparent rate constants of phenol degradation by CAP with photo-composite of different rGO ratios (calcinated at 400 °C for 2h). (**b**) Degradation of phenol by CAP in the presence of Ag/TiO_2_-rGO at different calcination temperatures. Solid lines represent the nonlinear curve fittings using the equation of 1st order kinetics. (**c**) Performance of 0.1% Ag/TiO_2_-1%rGO (calcined at 400 °C for 2 h) photo-composite in the presence of CAP on the reuse experiments.

The reaction kinetics of the combination of CAP with Ag/TiO_2_-rGO photo-composite demonstrates the rate of phenol removal. The degradation of phenol was described by the following rate equation:

2 $$\frac{{ - d\left[ {Ph} \right]}}{dt} = k_{app} \left[ {Ph} \right],$$

where $k_{app}$ ($min^{ - 1}$) is the apparent rate constant for the degradation of phenol, [Ph] is the concentration of phenol, and $t$ (min) is the treatment time. The nonlinear curve fitting shows that the order of the reaction kinetics is 1^st^ order as $R^{2}$ close to unity (Fig. 5b). Figure 5a presents the effect of the rGO ratio on phenol removal. It presented the apparent rate constants $k_{app}$($min^{ - 1}$) of phenol degradation by CAP with different photocatalysts. The $k_{app}$ of phenol in the case of CAP in the presence of photocatalysts is mainly higher than in the case of CAP only. The nano-hybrid photocatalyst that loaded with 1% wt rGO has the highest $k_{app}$. Figure 5a shows that the rGO has a synergistic effect with Ag and TiO_2_, this effect enhances the photocatalytic performance of Ag/TiO_2_ composite. This is attributed to the formation of a hybrid system that prolonged the lifetime of $e^{ - }$ and $h^{ + }$ followed by decreasing the charge separation rate. This enhances the formation of oxidative radicals and increases the phenol rate of degradation. rGO is also shifted TiO_2_ to redshift (as mentioned before), consequently, TiO_2_ has absorbed the whole light spectrum that generates from CAP. Increasing the ratio of rGO in the photo-composite led to smaller values of $k_{app}$ when compared to rGO (1 wt%)-TiO_2_. This behavior may be attributed to the stacking of rGO nanosheets when their loading is increased, which decreases the light interaction with nano-composite. Moreover, composites with rGO loadings above 1 wt% should display a highly negatively charged surface, which could decrease the separation of the photo-generated $e^{ - }$-$h^{ + }$ pairs and also result in a fast recombination rate of these charge carriers which consistent with^38^. Based on these results, it appears that 1 wt% is the optimal loading of rGO to obtain a close 0.1% Ag/TiO_2_-rGO interfacial contact, leading to both an effective activation of TiO_2_ by CAP radiation and an enhanced charge transfer between rGO and 0.1% Ag/TiO_2_.

Figure 5b shows the effect of calcination temperature (300 °C, 400 °C, and 500 °C) of 0.1% Ag/TiO_2_-1% rGO on phenol removal. The photo-composite **(**0.1% Ag/TiO_2_-1% rGO at 400 °C) has the highest removal rate due to the more reduction of GO at this temperature and shifting to the G band in Raman (see Fig. 1b, inset b). This leads to an increase in charge transfer between TiO_2_ and rGO and consequently the charge separation is increased and the recombination rate of $e^{ - }$/$h^{ + }$ pairs is decreased. On the other hand, the lowest removal rate in the case of calcination temperature of 500 °C could be due to the growth in grain crystal size of TiO_2_ (see XRD section), which decreases the surface area of the photo-composite and the active sites that generate $e^{ - }$/$h^{ + }$ pairs.

Reusing this photo-composite in the presence of CAP is an important issue for practical application in any future water treatment and an advantage making the treatment process cost-effective. Figure 5c shows the use of 0.1% Ag/TiO2-1%rGO (calcined at 400 °C for 2 h) for the degradation of different solutions of phenol several times. The photo-composite was recovered after each run by settling the photo-composite and decantation the treated water. The degradation efficiency of phenol (in terms of removal percentage at 30 min) was decreased to about 65% for up to four consecutive runs without cleaning the photo-composite. The removal of phenol increased back to about 100% at the fifth run after cleaning the photo-composite with distilled water and using it again. This can be due to blocking of some photo-composite active sites through adsorption of phenol byproducts, which decreases the number of generated $e^{ - }$/$h^{ + }$ pairs. These findings are in agreement with^39^. Insignificant change in the degradation efficiency of phenol was observed for up to five consecutive runs with cleaning the photo-composite between each use, indicating that photo-composite can conserve its photocatalytic efficiency and stability in the presence of CAP for a long time. Herewith, it is a promising stable photo-composite for long-term practical application with the CAP process.

### Microbial removal

#### In Vitro antimicrobial assay of 0.1% Ag/TiO_2_-1% rGO nanocomposite

The inhibition zone diameters were measured around the saturated discs by the synthesized 0.1% Ag/TiO_2_-1% rGO nanocomposite which was prepared at three temperatures (300 °C, 400 °C, and 500 °C) against different microbial pathogens (Figure S1 in the SI). The results exhibited that the prepared 0.1% Ag/TiO_2_-1% rGO nanocomposite at three temperatures has a potent antimicrobial effect against Gram-negative bacteria (*E. coli*, *Salmonella Typhimurium* and *Pseudomonas aeruginosa*) and Gram-positive bacteria (*Listeria monocytogenes*, *Staphylococcus aureus*, *Enterococcus faecalis* and *Bacillus subtilis*) and also against *Candida albicans* as a fungal strain. The inhibition zone diameters were slightly high with the prepared 0.1% Ag/TiO_2_-1% rGO composite at 500 °C against *Enterococcus faecalis* (13 mm), *Listeria monocytogenes* (12 mm), and *Candida albicans* (12 mm). TiO_2_ nanocomposites have been shown to exhibit antibacterial effects and effectively work in light and dark conditions for multipurpose environmental applications. Whereas, the inhibition effect of TiO_2_ NPs for the inactivation of microorganisms under dark conditions depends on the shape, size, and orientation of the nanocrystals^40^. The bacterial inactivation revealed electrons transfer from the bacterial membrane to the TiO_2_ surface followed by the interface due to the Schottky barrier effect^41^.

#### Effect of Ag/TiO_2_-rGO nanocomposite and non-thermal plasma on bacterial strains

**Figure 6:**
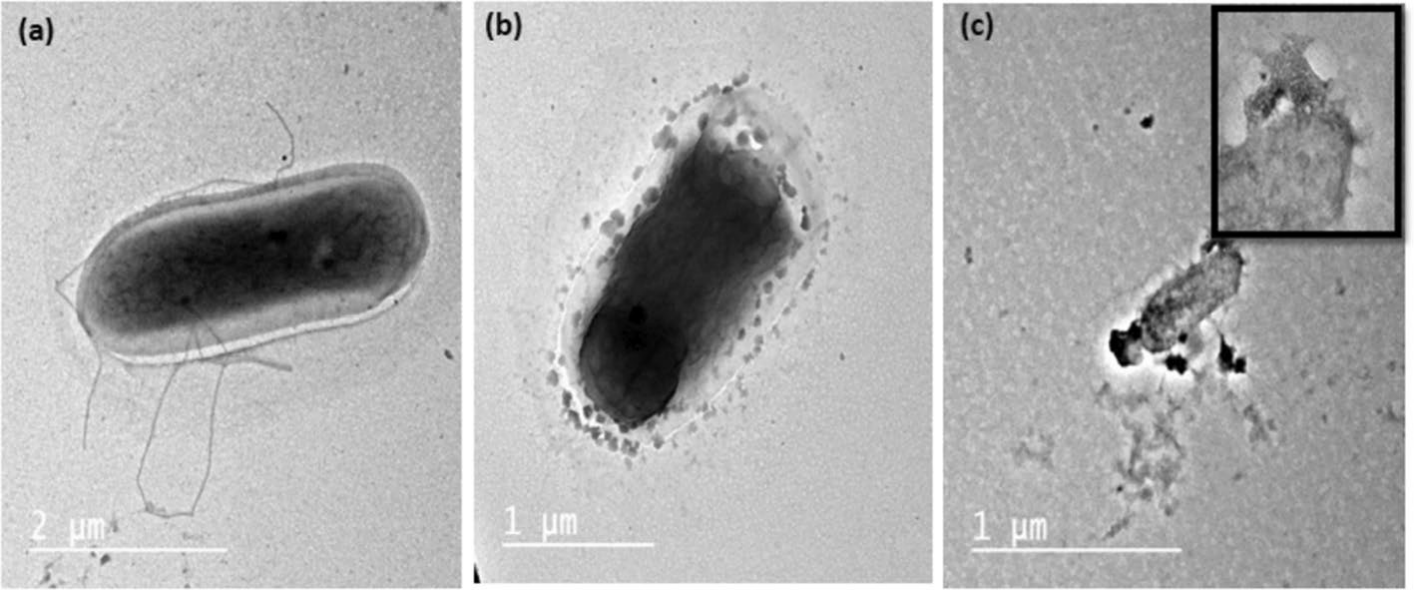
Antibacterial effect of CAP and 0.1% Ag/TiO_2_-1% rGO nanocomposite against *E. coli* (**a**) normal *E. coli* cell and its flagella (**b**) loss of membrane integrity after exposure to CAP, and (**c**) The destroyed *E. coli* cell with ruptured cell membrane and cytoplasm release after contact with 0.1% Ag/TiO_2_-1% rGO nanocomposite.

According to the bacterial inactivation results, it was not possible to determine the synergistic effect between CAP and NPs as complete inactivation (5log_10_ reduction) of both *E. coli* and *S. aureus* by NP under dark conditions (no CAP exposure) was observed (Table S2 in SI). However, we found that brief exposure to CAP promotes a rapid loss of cell membrane integrity (see Fig. 6a, b). Since CAP comprises both (V)UV and particles, it produces the most diverse stress on the cell and its components due to physical synergy reactions between (V)UV photons and particles. Despite the fact that UV radiation is the primary disinfection element in plasma disinfection, its direct effects on microbial cells are rather limited due to a lack of penetration^42^. Also, the antibacterial effect of 0.1% Ag/TiO_2_-1% rGO nanocomposite under dark conditions was further examined using TEM Fig. 6c, where incubation with nanocomposite under dark conditions result in visible rupture and leakage of cytoplasmic contents of *E. coli* which could be attributed to the combination between Ag and rGO nanocomposite leading to scratching of the bacterial cell membrane^43^.

### Removal of viruses

**Figure 7:**
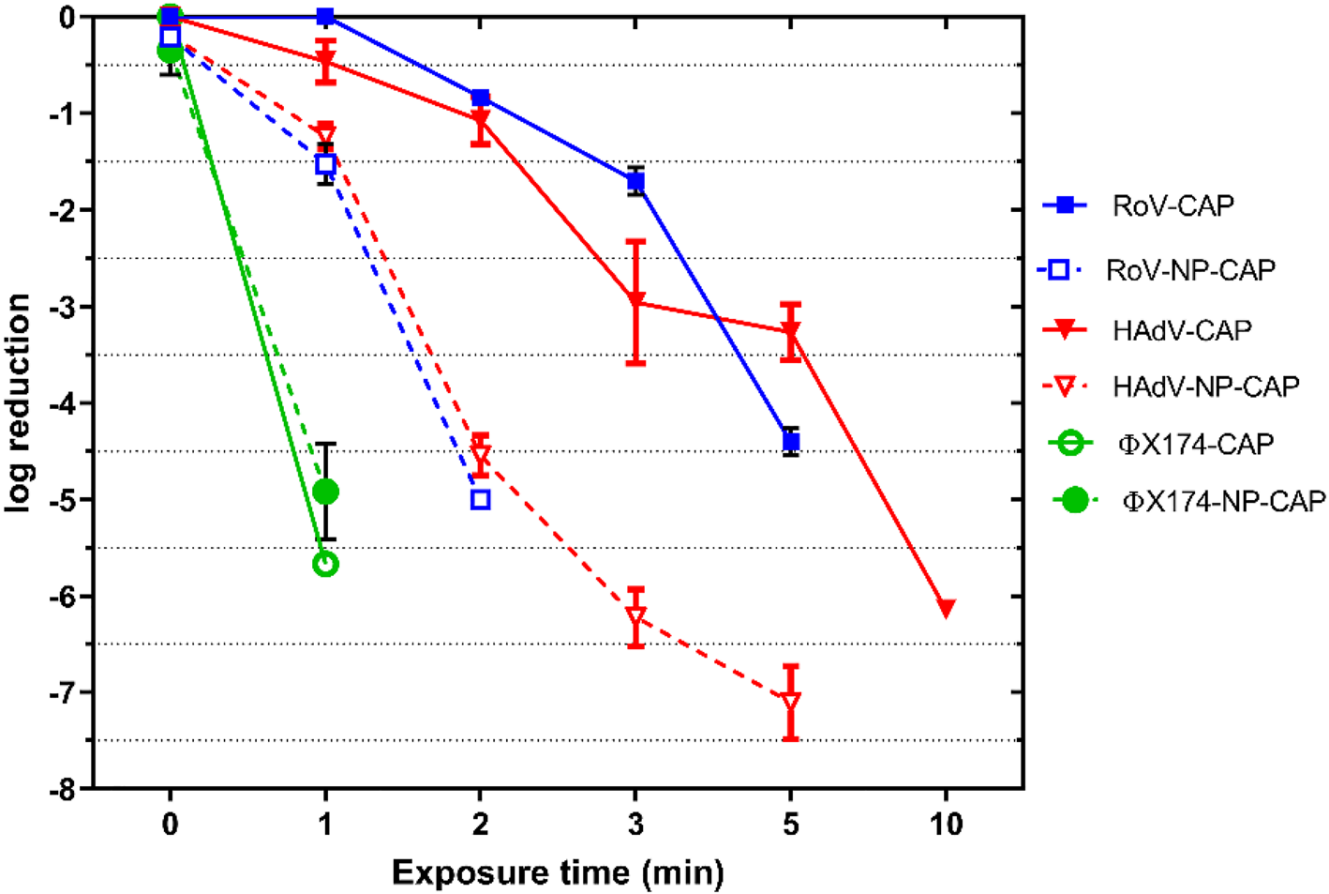
Inactivation of viruses suspended in distilled H_2_O by CAP and CAP-0.1% Ag/TiO_2_-1% rGO photo-composite. Zero time (no CAP) exposure is the dark inactivation. Results are an average of duplicate experiments and the error bars indicate the SEM.

CAP showed virucidal action against ɸX174, achieving a 5.5 log_10_ decrease in 1 min and a 4.3 log_10_ removal of RoV in 5 min (Fig. 7). However, AdV was more resistant to the plasma treatment as complete inactivation (~ 6log_10_) reduction was achieved after 10 min exposure to CAP (Fig. 7). Interestingly, the time required by CAP to inactivate viruses was reduced by using 0.1% Ag/TiO_2_-1% rGO. For example, Fig. 7 shows that at 2 min of CAP exposure 0.8 log_10_ reduction of RoV was observed but with CAP treatment in the presence of 0.1% Ag/TiO_2_-1% rGO a complete inactivation of RoV at the same exposure time was found. Also, using CAP with 0.1% Ag/TiO_2_-1% rGO resulted in > 4.7log_10_ reduction of AdV at 2 min treatment, whereas < 1log_10_ could be reduced using only CAP at the same exposure time. More importantly, dark treatment (0.1% Ag/TiO_2_-1% rGO without CAP) showed non-significant removal of viruses after 10 min exposure (0.1–0.5 log_10_) which could be due to adsorption to the 0.1% Ag/TiO_2_-1% rGO and inactivation by the small percentage of Ag.

Indeed, the high antiviral effect of CAP might be due to the chemical interactions between viral components and ROS and RNS. Peroxidation of the protein and reduction of infectivity can occur when active species interact with viral capsid protein. Furthermore, reactive species can cause damage to viral nucleic acid, resulting in impaired gene expression or full nucleic acid destruction. Plasma exposure may produce viral DNA-DNA crosslinking or DNA–protein complexes, causing bacteriophage DNA and protein damage. The inactivation of ɸX174 after short exposure to plasma could be attributed to the damage to the viral capsid. It has been found that the effect of plasma on bacteriophage lambda primarily resulted mainly from the damage of the viral capsid protein and only a less degree of damage to viral DNA. Using qPCR, only 0.4log_10_ and 1.1log_10_ removal of AdV was found by CAP and 0.1% Ag/TiO_2_-1% rGO with CAP, respectively in 10 min exposure (data not shown).

Direct incubation with NP without CAP was responsible for a reduction of ~ 0.12log_10_, ~ 0.2log_10_ and 0.5log_10_ of AdV, RoV and ɸX174, respectively. This minimal effect of dark inactivation was consistent with Liga et al.,^16^ who found a partial reduction of MS2 after incubation with nAg/TiO_2_ due to interactions of viral capsid amino acids with silver. However, it should be noted that in the present study 0.1% Ag/TiO_2_-1% rGO contains a small amount of Ag.

The enhanced inactivation by NP was also observed after CAP exposure (Fig. 7) due to the synergetic effect of CAP on photocatalytic oxidation activity of TiO_2_ instead of the antimicrobial property of nAg. Furthermore, silver doping in TiO_2_ has been found to promote charge separation, leading to more effective ROS production and, as a result, increased viral inactivation. Whereas, the degree of viral inactivation depends primarily on the number of viruses adsorbed on the TiO_2_ surface^44^.

### The suggested CAP-Ag/TiO_2_-1% rGO action mechanism

**Figure 8:**
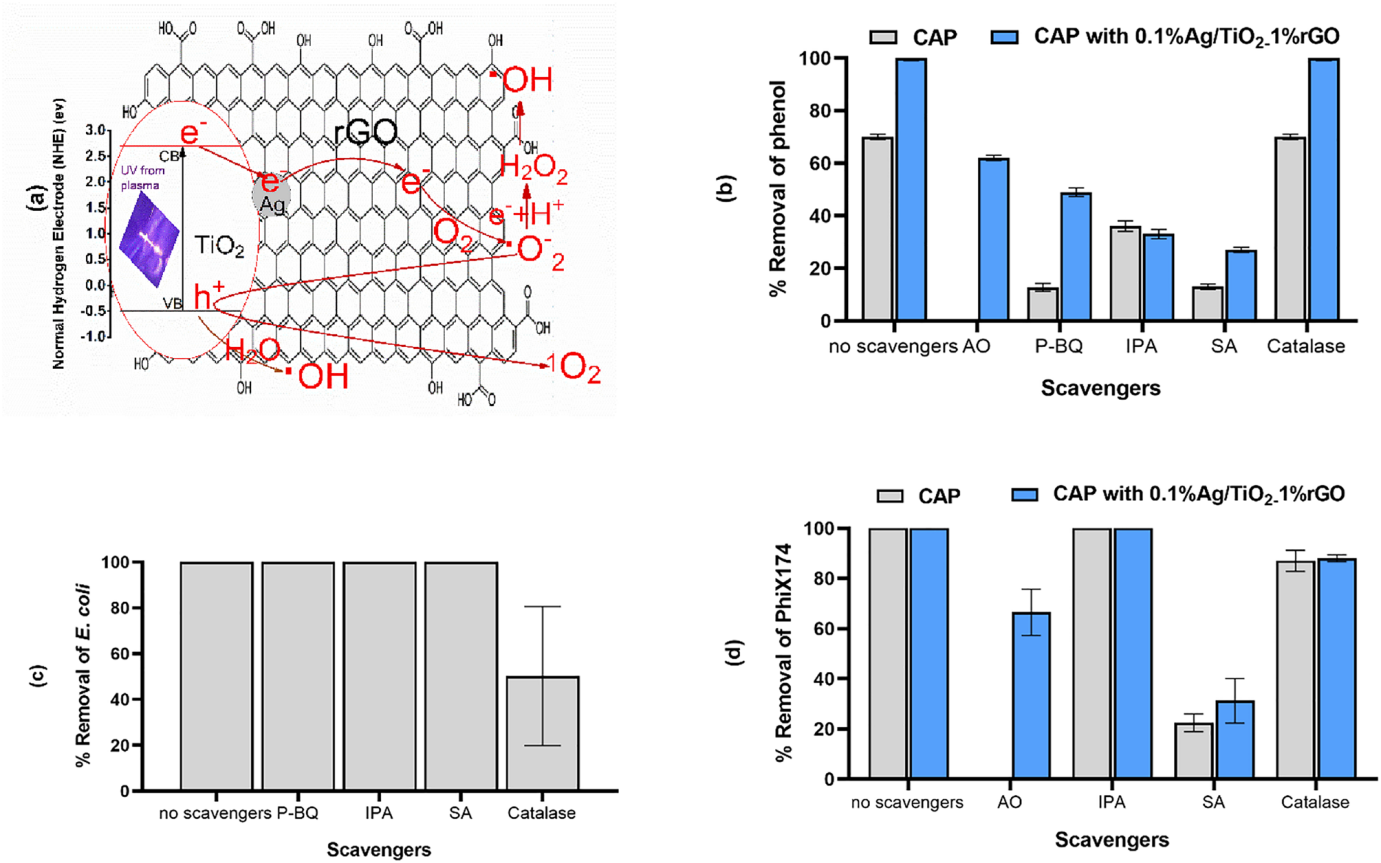
(**a**) Suggested photocatalytic mechanism for phenol degradation by CAP-Ag/TiO_2_-1% rGO photo-composite. Effect of scavengers on the removal efficiency of phenol (**b**), *E. coli* (**c**) and Phix174 (**d**). Data are an average of duplicate batch experiments and the error bars indicate the SEM.

A mechanism describing the degradation of phenol using Ag/TiO_2_-1% rGO photo-composite in the presence of CAP was suggested based on the results of phenol degradation and generated carriers scavenger experiments (Fig. 8). The produced UV light from CAP is capable of activating the TiO_2_ in the photo-composite. Figure 8a presents the transfer process of excited charge carriers, where the generated electrons transferred from the valence band (VB) to the conduction band (CB). The formed $h^{ + }$ react with the adsorbed H_2_O and OH^−^ on the surface of the composite to generate ^•^OH radicals. On the other hand, $e^{ - }$ transfer from CB to Ag then to rGO reduces the rate of $e^{ - }$/$h^{ + }$ recombination and enhances the photocatalytic activity of the photo-composite. $e^{ - }$ react with dissolved oxygen to produce superoxide radicals (O_2_^•−^), which react with $h^{ + }$ and produce singlet oxygen (^1^O_2_) species. Also, ^•^O^-^_2_ react with $e^{ - }$ and H^+^ to form hydrogen peroxide (H_2_O_2_), which can form ^•^OH by the action of UV light. In addition to the CAP production for ROS and RNS, this system has a cocktail of radicals. Therefore and for simplicity, instead of following up the formation of these radicals, the contribution of most formed radicals can be determined using radical scavengers.

The role of the reactive species generated in the CAP-0.1% Ag/TiO_2_-1% rGO process was investigated by using ammonium oxalate (AO), para-benzoquinone (p-BQ), sodium azide (SA), isopropyl alcohol (IPA), and catalase to trap $h^{ + }$, O_2_^•−^, ^1^O_2_, ^•^OH, and H_2_O_2_ respectively^10,45,46^. Phenol degradation using CAP only or CAP-0.1% Ag/TiO_2_-1% rGO in presence of scavengers is shown in Fig. 8b. In CAP only, the %removal of phenol decreased after adding p-BQ, IPA, and SA indicating that O_2_^•−^, ^•^OH, and ^1^O_2_, respectively, have a major role in the phenol degradation by CAP only and the contribution of oxidizing species in the phenol degradation process follows the order: ^1^O_2_ = O_2_^•−^  > ^•^OH. There was no effect of H_2_O_2_ as the %removal of phenol did not change by adding catalase in case of CAP only or CAP-0.1% Ag/TiO_2_-1% rGO. The additional role of $h^{ + }$, ^1^O_2_, and ^•^OH appeared by using the photo-composite and the contribution of these oxidizing species follows the order: ^1^O_2_ > ^•^OH > O_2_^•−^  > $h^{ + }$. Singlet oxygen, therefore, plays the most important role in the phenol degradation in this process.

Recently, it was also proposed that adding graphene nanomaterials to TiO_2_ could enhance its antibacterial activity in darkness this might explain the complete inactivation of bacteria by Ag/TiO_2_-1% rGO photo-composite without CAP exposure^47^. Additionally, under the conditions described here, in the case of CAP treatment without photo-composite, H_2_O_2_ showed a high contribution to bacterial cell damage as has been investigated using catalase as H_2_O_2_ scavenger (Fig. 8c). Similarly, the discharge of H_2_O_2_ in plasma-activated water was shown to significantly contribute to bacterial inactivation before^48^. However, other reports found that CAP produced O and OH species that could either react with the bacterial outer membrane or easily penetrate the bacteria cells and damage them^49^.

The present study showed that ^1^O_2_ has a relatively high contribution to viral inactivation by CAP (Fig. 8d) within 1 min exposure. An identical course of action has been suggested in other reports. According to GuO et al.,^50^, bacteriophages can be inactivated by plasma through several actions of singlet ^1^O_2_. Additionally, the inactivation of feline calicivirus (FCV) and bacteriophages T4 was shown to be most effective by ^1^O_2_ which could alter the molecular mass of methionine and oxidizing histidine residues^51^. Here, we found that ^1^O_2_ and $h^{ + }$ have significant contribution in viral inactivation by CAP-Ag/TiO_2_-1% rGO photo-composite (Fig. 8d), as the viral removal was decreased in the presence of SA (scavenger to ^1^O_2_) and AO which trap the $h^{ + }$. The $h^{ + }$ induce the oxidation processes and therefore various ROS are created. ROS emerging on the photocatalytic surfaces inactivates infectious agents including viruses^52^. Rather than full destruction of the capsid or nucleic acid, certain amino acids on capsid proteins could be oxidized, changing their affinity to host cell receptors. Also, the effect of H_2_O_2_ on viral inactivation was low which might be due to the use of non-enveloped viruses. This observation is consistent with other reports which found that the effect of H_2_O_2_ on naked viruses such as AdV and FCV is a secondary effect^53,54^.

## Conclusions

This study demonstrated that photo-composite of 1% rGO ratio has the best phenol degradation rate in aqueous solution in the presence of CAP, compared also to pure TiO_2_ and Ag/ TiO_2_ photo-composite. The rGO has a synergistic effect with Ag and TiO_2_, this effect enhanced the photocatalytic performance of the Ag/TiO_2_ composite by the formation of a hybrid system that prolonged the lifetime of $e^{ - }$ and $h^{ + }$ followed by decreasing the charge separation rate. It was found that the photo-composite 0.1% Ag/TiO_2_-1% rGO at 400 °C has the highest efficiency towards the phenol degradation in the presence of CAP. This could be attributed to the more reduction of GO at this temperature which leads to an increase in charge transfer between TiO_2_ and rGO and consequently decreases the recombination rate of $e^{ - }$/$h^{ + }$ pairs. The study found that the most important role in the phenol degradation was played by singlet oxygen in this process. Moreover, data revealed that 0.1% Ag/TiO_2_-1% rGO at 400 °C is a promising stable photocatalyst for long-term application in the presence of CAP process, ensuring the sustainability of the technology. Further, the photocatalytic microbial inactivation tests of bacteria and enteric viruses demonstrated excellent performance. Accordingly, 0.1% Ag/TiO_2_-1% rGO nanoparticles appear to be a promising candidate for environmental applications. Further research is needed to address the impact of water quality on the performance of composite nanoparticles in the presence of CAP treatment.

## Material and methods

### Synthesis of Ag/TiO_2_-rGO photo-composite

GO was prepared according to the modified Hummers method^8^ and Ag/TiO_2_ was prepared according to Badawy et al.^22^. The details of their preparation were described in the SI. To form 0.1 Ag/TiO_2_-GO, the GO suspension was dried at 80 °C with a mixture of titanium peroxide sol with the reduced AgNO_3_ solution in a rotary evaporator. This drying step is to allow strong attachment of Ag NPs and GO with titanium dioxide matrix (more details are in SI). The produced 0.1 Ag/TiO_2_-GO powder was thermally treated at 400 °C to reduce the GO and crystallize TiO_2_ simultaneously. The doping ratios of rGO was ranging from 0.5% to 5% rGO to Ag/TiO_2_. Finally, the effect of the calcination temperature was studied by heating the optimum doping ratio at 300 °C, 400 °C, and 500 °C.

### Characterization of the prepared materials

The phase composition of the prepared materials was studied by X-ray diffraction (XRD) using diffractograms collected by PANalytical X'Pert Pro diffractometer with CuKα source (λ = 1.5406 Å). The morphology of the 0.1% Ag/TiO_2_-1% rGO photo-composite was investigated by high-resolution TEM model JEM 2100-HRTEM (JEOL, USA, Inc.) operated at an accelerating voltage of 200 kV. Raman spectra were acquired using a WITec Alpha 300 RA confocal Raman microscope (WITec GmbH, Ulm, Germany). The ultra violet-visible light (UV–Vis) diffuse reflectance spectra (DRS) were measured by a spectrometer (JASCO, Model V730, Japan) equipped with diffuse reflectance accessories, using BaSO_4_ as the reference sample,) and the photoluminescence (PL) was determined by spectrofluorometer (JASCO, Model FP-6500, Japan) at excitation wavelength 315 nm. The light source was a Xenon arc lamp (150 Watt).

### Antimicrobial assay of 0.1% Ag/TiO_2_-1% rGO photo-composite by disc diffusion

The antimicrobial activity of 0.1% Ag/TiO_2_-1% rGO photo composite using disc diffusion assay was carried out for different Gram-negative, Gram-positive bacteria, and *Candida albicans* as described before^55^, more details can be found in SI.

### Examination of *E. coli* by transmission electron microscopy

The effect of 0.1% Ag/TiO_2_-1% rGO photo-composite on *E. coli* was investigated. Approximately, 10 mL of ~ 10^5^ CFU/mL fresh *E.coli* suspensions with and without 1 mg/mL of 0.1% Ag/TiO_2_-1% rGO photo-composite was prepared for TEM examination. One drop of each suspension was placed on a glow discharged formvar coated copper grid for a minute. The excess suspension was absorbed using filter paper, and the preparation was dried for 5 min in air. The samples were examined using high-resolution TEM.

### The cold atmospheric plasma system

A high voltage probe (P5101) with a division ratio 1/1000 was connected to the plasma electrodes to measure the plasma voltage during the treatment and a digital multimeter (Sanwa Cd770) was used to measure the current in the circuit during the treatment. A digital oscilloscope (25 MHz) was used for monitoring the electrical parameters. The OES of the plasma was measured by a spectral measurement system (SMS-500) with a wavelength accuracy of ± 0.25 nm**.**

### Phenol degradation

The performance of the prepared photocatalysts towards the degradation of phenol (Sigma-Aldrich) was investigated under CAP. For this purpose, phenol was selected as a model of chemical water pollutant. For the degradation experiments, 10 mL of 50 mg/L phenol solutions were placed in a glass Petri dish with internal diameters of 5.6 cm to have a water thickness of 3.6 mm. These are the optimum operation conditions, as mentioned before, that were determined in our previous work^2^. 10 mg of the prepared photocatalysts were magnetically stirred and filtered by a 0.2 μm syringe-driven filter unit (Thermo Fisher Scientific) before analysis. The experiments were performed in the batch system and the removal of phenol at definite treatment times was determined using high performance liquid chromatograph (HPLC, Agilent 1260, USA). The program analysis of the HPLC can be found in SI. Each point was measured triplet and the average was recorded. Moreover, nitrates and nitrites measurements were done by Ion chromatography (ICs 5000).

### Effect of 0.1% Ag/TiO_2_-1% rGO photo-composite and CAP on bacterial strains

*E. coli* (2.6 × 10^5^ CFU/mL) and *S. aureus* (1.0 × 10^5^ CFU/mL) suspensions were inoculated in 10 mL of sterile distilled water. Each bacterial strain was directly exposed to CAP and another set was subjected to CAP in the presence of 10 mg/10 mL of 0.1% Ag/TiO_2_-1% rGO photo-composite. The bacterial strains were determined before and after treatment by tenfold serial dilution using the pour plate method. The colonies were expressed as a colony-forming unit (CFU/mL). All CAP or NPs treatments were conducted in duplicates batch experiments and bacterial quantification was performed.

### Viruses and plasma treatment

#### Virus inactivation experiments

Nanoparticles suspension was freshly prepared in dH_2_O at a concentration of 1 mg/mL. Ten mL of the NPs suspension was spiked with ɸX174, AdV5, and RoV SA11 separately and mixed thoroughly within the reactor. The mixture was exposed to CAPs at different time courses (1, 2, 3, 5, and 10 min), and adjusted plasma voltage at approximately 3 kV with a gap distance of 1 mm. In order to know differences in viral inactivation, control groups were included for the comparison: (i) one set was incubated at dark without CAP, (ii) one set was subjected to plasma without NPs, and (iii) control group of viruses without neither NPs nor CAP.

Samples containing NPs were counted immediately or after centrifugation at 5000 *xg* for 10 min to remove the NPs and see if the presence of NPs interferes with viral enumeration. There was no clear difference between the two measures. So, the results presented here were acquired from direct enumeration of the samples without the removal of the nanoparticles. All CAP or CAP with NPs treatments were conducted in duplicates batch experiments and virus quantification was performed as explained below.

### Model viruses

#### Somatic coliphages

ɸX174 was enumerated in water samples by double agar layer plaque test according to ISO 10705–2. *E. coli* DSM 13127 grown on tryptone broth was used as a host. In each assay one tube with 1 mL tryptone broth test tube was used as negative control and another tube with ~ 50PFU/ml of ɸX174 as a positive control.

#### Adenovirus

In order to examine the infectivity of Adenovirus type 5 in the treated water samples, the integrated cell culture qPCR (ICC-qPCR) assay has been used according to Hamza et al.^57^. Viral DNA extraction was performed by QIAamp DNA Mini Kit. SYBR Green qPCR was conducted to determine AdV copy numbers in the treated and control samples. QPCR was performed in a 20 µL reaction volume containing 5 µL of nucleic acid template and 0.25 µM each of forward and reverse AdV primers (AQ1 5′-GCCACGGTGGGGTTTCTAAACTT-3′ , AQ2 5′-GCCCCAGTGGTCTTACATGCACATC-3′) according to Heim et al.^58^. The reaction temperature was 95 °C for 10 min, and 45 cycles of 95 °C for 15 s followed by 60 °C for 1 min. Amplification, detection, and analysis were done by Rotorgene 6000 real time PCR system (Corbett Research, Sydney, Australia).

#### Rotavirus

Rotavirus SA11 was used as a model to RNA enteric viruses. Viral stock was prepared by propagation on MA-104 cells as described before^2^. Virus titration was performed by the end-point dilution assay TCID_50_. In order to quantitatively assess RoV in treated and non-treated water samples, the infected cells were examined under the microscope for the development of CPE, and the virus concentration was expressed as TCID_50_/mL.

### Trapping of reactive species

The scavengers are used to investigate the role of reactive species generated in the CAP-0.1% Ag/TiO_2_-1% rGO process. AO, p-BQ, SA, IPA, and catalase are capable of trapping $h^{ + }$, O_2_^•−^, ^1^O_2_, HO^•^, and H_2_O_2_ respectively. All quenchers with a concentration of 100 mM except catalase (40 µg/10 mL) were added individually to the phenol solution or *E. coli* or Phix 174 and the CAP-photo-composite experiments were performed as mentioned previously. However, p-BQ displayed antiviral activity, therefore the role of O_2_^•−^ in viral inactivation could not be studied.

## Supplementary Information

Supplementary Information.

## Data Availability

All data generated or analyzed during this study are included in this published article and its supplementary information file.
