# Supplementary material for: Removal of chemical and microbial water pollutants by cold plasma combined with Ag/TiO2-rGO nanoparticles
Source: Sci Rep. 2022 Jun 14;12:9850. doi: 10.1038/s41598-022-13444-2 (PMC9198087; doi:10.1038/s41598-022-13444-2)
Supplement: Supplementary file 1 — Supplementary Information. [file 41598_2022_13444_MOESM1_ESM.docx]

**Supporting Information**

**Removal of chemical and microbial water pollutants by cold plasma combined with Ag/TiO_2_- rGO nanoparticles**

Mahmoud S. Abdel-Wahed, Mohamed Mokhtar Hefny, Sherif Abd-Elmaksoud, Mohamed A. El-Liethy, Marwa A. Kamel, Amer S. El-Kalliny, Ibrahim Ahmed Hamza

**Synthesis of graphene oxide (GO)**

9 g of KMnO_4_ (Fisher Scientific Co.) was added slowly to a mixture of 150 mL of conc. H_2_SO_4_ (Fisher Scientific Co.), 2 g of graphite (Fisher Scientific Co.) and 1.5 g of NaNO_3_ (Bio Basic Canada INC.) within 20 min under strong stirring in an ice-water bath. This was followed by stirring at 35 °C for 2 h and then, 90 mL of deionized water (DW) was added under vigorous stirring at 95 °C for 15 min. Then, 10 mL of 30% H_2_O_2_ (Sigma-Aldrich Chemicals company) was added to get rid of residual KMnO_4_. The produced bright yellow suspension was washed by 3% HCl and DW until the pH reached 5 – 6 in order to remove the excessive metal ions. The separation of the produced GO was done by centrifugation (MEGAFUGE 16, Thermo Fisher Scientific) at 13000 rpm for 1 h. The final GO product was dried for 24 h at 50 °C under vacuum condition in an oven (DAIHAN Scientific MOV-30).

**Synthesis of 0.1 Ag/TiO_2_­- rGO**

Cetyltrimethylammonium bromide (CTAB) was used as template and hydrazine hydrate as reducing agent. 0.1% Ag/TiO_2_ was prepared by mixing 0.37 mL of 1 mM aqueous hydrazine solution and 14.8 mL of 1 mM CTAB aqueous solution by stirring for 1 min at room temperature. Then, 0.37 mL of 50 mM aqueous AgNO_3_ solution was added to the mixture. The formed dark brown solution was further stirred for another 10 min to complete the redox reaction. This solution was called Solution (A).

On the other hand, hydrolysis of 7.7 mL titanium tetraisopropoxide (TTIP) was done by adding 425 mL DW to form titanium hydroxide precipitate. The precipitate was rinsed many times with DW to remove the formed alcohol from hydrolysis step. Afterward, 85.3 mL of 15% H_2_O_2_ was added slowly to the precipitate till complete dissolution of the precipitate forming titanium peroxide sol. This was named Solution (B). The two solutions A and B were mixed completely and named solution (C).

Ag/TiO_2_- rGO was prepared by suspending 100 mg of GO in 100 mL DW by sonication for 30 min. The suspension was mixed with solution C by sonication for 5 min. Afterward, the formed suspension was dried at 80 ºC in rotary evaporator (Heidolph, Germany) to allow strong attachment of Ag NPs and GO to titanium dioxide matrix.

**HPLC conditions**

The HPLC is equipped with the analytical column Zorbax reversed phase C18 (4.6 mm ID × 250 mm, 5 μm particle size) and a diode-array detector, at 280 nm wavelength. The column temperature was kept at 25 °C during the analysis. Gradient elution was carried out using water (mobile phase A) and acetonitrile (mobile phase B). The 75% A mobile phase was eluted for 1 min, then decreased to 60% A for 2 min. The flow rate of the mobile phase was kept at 0.5 mL/min.

***In* *vitro* antimicrobial assay of 0.1% Ag/TiO_2_- 1% rGO nanocomposite**

The antimicrobial activity of 0.1% Ag/TiO_2_- 1% rGO photo composite using disc diffusion assay was carried out. *Escherichia coli* (ATCC 25922), *Salmonella* *typhimurium* (ATCC 14028), *Pseudomonas aeruginosa* (ATCC 10145) as Gram-negative bacteria and *Listeria monocytogenes* (ATCC 25152), *Staphylococcus aureus* (ATCC 43300), *Enterococcus faecalis* (ATCC 43845), and *Bacillus subtilis* as Gram-positive bacteria and *Candida albicans* as a mold were used in this study. 24 h fresh microbial strain suspension was spread onto Müller Hinton agar plate (BBL™, Germany). Sterile discs were soaked in the 0.1% Ag/TiO_2_- 1% rGO nanocomposite suspensions (that prepared at three temperatures; 300, 400 and, 500^○^C). The soaked discs were placed onto the inoculated Müller Hinton agar plates. All plates were incubated at 37 °C for 24 h. The inhibition zone diameters were measured in mm using measuring ruler.

**Results**

**Plasma electron temperature**

**Table S1** Spectroscopic data of N II ^1^

| \| **λ (nm)** \| Spectrum \| E_exc_(cm^-1^) \| g_m_.A_m_ (s^-1^) \| \| --- \| --- \| --- \| --- \| | | | |
| --- | --- | --- | --- | --- | --- | --- | --- |
| 393.04 (λ_0_) | N II | 228 694.30 | 2650000 |
| 399.4997 | N II | 174 212.03 | 6.10E8 |
| 439.3847 | N II | 189 335.16 | 365000 |
| 448.8095 | N II | 188 857.37 | 4.31E6 |
| 456.476 | N II | 186 511.58 | 8.25E6 |
| 465.4531 | N II | 170 666.23 | 9.60E6 |

**Effect of Ag/TiO_2_-rGO nanocomposite and non-thermal plasma on bacterial strains**

**Table S2.** Log reductions of the tested bacterial strains by exposure to CAP alone and CAP-NP (CAP-Ag/TiO_2_-rGO)

| **Time**  **(min)** | ***E. coli*  (CFU/mL)** | | | | ***S. aureus* (CFU/mL)** | | | |
| --- | --- | --- | --- | --- | --- | --- | --- | --- |
|  | **CAP** | | **NPs** | | **CAP** | | **NPs** | |
|  | **mean counts** | **Log reduction** | **mean counts** | **Log reduction** | **Mean counts** | **Log reduction** | **Mean counts** | **Log reduction** |
| **Zero** | 1.0x10^7^ | --- | 2.6x10^5^ | --- | 1.1x10^7^ | --- | 1.0x10^5^ | --- |
| **0.5 min** | 6.0x10^2^ | 4.2 | 0 | 5.4* | 1.0x10^3^ | 4.0 | 0 | 5.0* |
| **1 min** | 90 | 5.0 | 0 | 5.4 | 90 | 5.0 | 0 | 5.0 |
| **2 min** | 85 | 5.0 | 0 | 5.4 | 75 | 5.1 | 0 | 5.0 |
| **3 min** | 77 | 5.1 | 0 | 5.4 | 50 | 5.3 | 0 | 5.0 |
| **5 min** | 70 | 5.1 | 0 | 5.4 | 40 | 5.4 | 0 | 5.0 |
| **10 min** | 50 | 5.3 | 0 | 5.4 | 9 | 6.0 | 0 | 5.0 |
| **15 min** | 37 | 5.4 | 0 | 5.4 | 0 | 7.0* | 0 | 5.0 |
| **20 min** | 0 | 7.0* | 0 | 5.4 | 0 | 7.0 | 0 | 5.0 |

ND: Not Detected

* complete inactivation (100% removal)

**Figure S1. Inhibition zone diameters in millimeter (mm) of the tested Ag/TiO_2_- rGO nanocomposite against microbial pathogens.**

References

1 Kramida, A., Ralchenko, Yu., Reader, J. and [NIST ASD Team](https://physics.nist.gov/PhysRefData/ASD/index.html#Team) (2021). *NIST Atomic Spectra Database* (version 5.9), [Online]. Available: <https://physics.nist.gov/asd> [Sat Apr 02 2022]. National Institute of Standards and Technology, Gaithersburg, MD. DOI: <https://doi.org/10.18434/T4W30F>
